# Management of Severe Dentoalveolar Trauma Following a Motorcycle Accident With Immediate Ridge Preservation and Delayed Implant Rehabilitation: A Case Report

**DOI:** 10.7759/cureus.116492

**Published:** 2026-09-18

**Authors:** Norby Rodriguez

**Affiliations:** 1 Dentistry, Prisma Odontologia Estetica S.A.S., Cali, COL

**Keywords:** alveolar ridge preservation, bone graft, collagen membrane, dental implants, dentoalveolar trauma, implant rehabilitation, interdisciplinary care, motorcycle accident, oral trauma, tooth extraction

## Abstract

Severe dentoalveolar trauma resulting from high-energy motorcycle accidents presents significant clinical challenges because of the combined injuries to the teeth, alveolar bone, and surrounding soft tissues and the potential for associated injuries beyond the oral cavity. This case report describes the staged management of a healthy male patient who presented following a motorcycle accident. Clinical and radiographic examinations revealed multiple non-restorable teeth involving the maxillary and mandibular anterior regions, accompanied by traumatic alveolar fractures and associated bone defects. Initial dental management included extraction of the non-restorable teeth, thorough irrigation with sterile saline, conservative debridement of nonviable fractured alveolar bone while preserving viable residual bone, and immediate ridge preservation using a bovine xenograft covered with a resorbable collagen membrane to preserve the alveolar ridge architecture for future implant rehabilitation. The patient received postoperative antibiotic and anti-inflammatory therapy. Given the high-energy mechanism of injury and the possibility of associated injuries beyond the oral cavity, prompt medical evaluation was recommended. Following uneventful healing and adequate maturation of the preserved ridges, dental implants were placed as part of a staged rehabilitation protocol. After the osseointegration period, the patient progressed to the prosthetic phase. Following second stage surgery and peri-implant soft tissue maturation, definitive implant-supported restorations were delivered, restoring esthetics, function, and occlusal relationships. Clinical and radiographic evaluations supported progression through the successive treatment phases, and the patient reported satisfaction with the final functional and esthetic outcome. This case illustrates a staged approach to severe dentoalveolar trauma involving careful assessment of traumatic injuries, preservation of viable alveolar structures, immediate ridge preservation in appropriately selected sites, and delayed implant-supported rehabilitation. In patients presenting after high-energy trauma, clinicians should also recognize the possibility of concomitant injuries and consider prompt medical evaluation before definitive dental management.

## Introduction

Traumatic dentoalveolar injuries resulting from high-energy motorcycle accidents may involve multiple tooth fractures, injury to the tooth-bearing alveolar process, and surrounding soft tissue damage [1-4]. These injuries present significant functional and esthetic challenges and require careful assessment to determine tooth restorability, the extent of alveolar injury, soft tissue involvement, and the appropriate timing of definitive dental treatment. Delayed or inadequate management may contribute to excessive ridge resorption, compromised implant placement, and less predictable restorative outcomes [5-8].

In patients presenting after high-energy motorcycle trauma, the initial assessment should extend beyond the oral cavity. Associated craniofacial, neurological, cervical, or systemic injuries may require prompt medical evaluation and should take priority over definitive dental treatment when clinically indicated. Dentists should therefore recognize the need for appropriate emergency medical referral before proceeding with definitive management of dentoalveolar injuries when potentially significant associated trauma has not been excluded [9,10].

Once the patient's overall condition permits dental treatment, management of the dentoalveolar injuries should focus on preserving viable hard and soft tissues and establishing favorable conditions for subsequent rehabilitation. In selected traumatic extraction sites, immediate alveolar ridge preservation may be considered following thorough irrigation, removal of nonviable tissue, and assessment of the remaining alveolar structures and soft tissue coverage. Particulate bone grafts combined with resorbable collagen membranes may help preserve ridge architecture and facilitate subsequent implant placement [11-17].

This report describes the staged management of a 24-year-old man who sustained severe maxillary and mandibular dentoalveolar trauma following a motorcycle accident. The dental treatment involved assessment of the traumatic injuries, extraction of non-restorable teeth, conservative debridement, immediate ridge preservation with a bovine xenograft and resorbable collagen membrane, delayed implant placement, and definitive implant-supported rehabilitation. Given the high-energy mechanism of injury, prompt medical evaluation was also recommended to assess for potentially associated injuries beyond the oral cavity. This case illustrates clinical considerations in the staged management and rehabilitation of severe dentoalveolar trauma [18-20].

## Case presentation

A 24-year-old man classified as American Society of Anesthesiologists (ASA) Physical Status I presented to the dental office after sustaining severe facial trauma in a motorcycle accident. The patient reported immediate pain, multiple fractured teeth, and difficulty chewing. Clinical examination revealed extensive dentoalveolar trauma with multiple non-restorable crown root fractures involving the maxillary right central incisor through the left first premolar (teeth #8-12) and the mandibular left central incisor through the left first premolar (teeth #21-24). Associated fractures of the alveolar bone were also identified. Following the clinical examination, radiographic assessment, including cone beam computed tomography (CBCT), was performed to evaluate the dentoalveolar injuries and associated alveolar bone defects (Figure 1).

**Figure 1:**
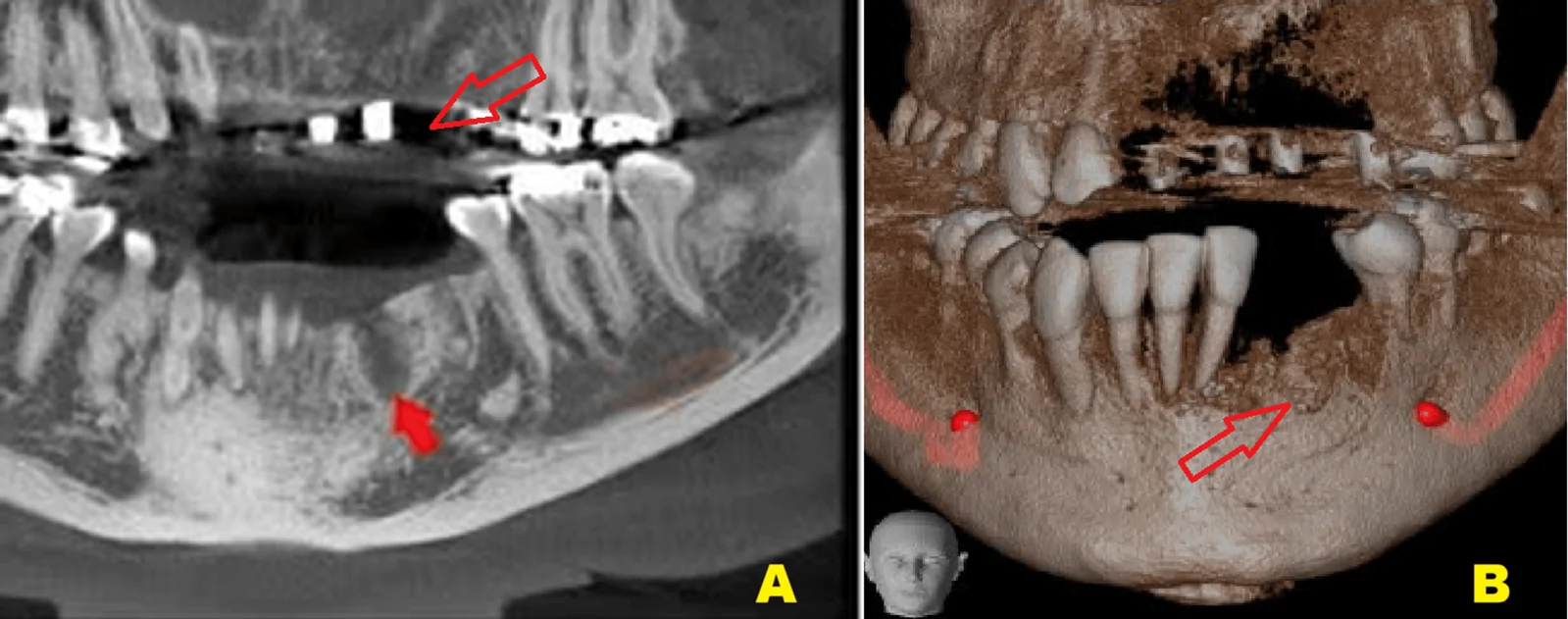
CBCT evaluation following the motorcycle accident. (A) Coronal CBCT image demonstrating dentoalveolar trauma involving the maxillary and mandibular anterior regions. The open arrow indicates the maxillary dentoalveolar region, and the solid arrow indicates the mandibular alveolar bone fracture. (B) Three-dimensional CBCT reconstruction demonstrating the maxillary and mandibular anterior dentoalveolar regions and associated alveolar bone defects. The open arrow indicates the mandibular traumatic defect. CBCT: cone beam computed tomography.

Following clinical and radiographic assessment, emergency surgical treatment was performed under local anesthesia. All non-restorable teeth were atraumatically extracted, and the surgical sites were thoroughly irrigated with sterile saline solution. Fractured and nonviable alveolar bone fragments were carefully debrided while preserving healthy bone whenever possible. Because of the traumatic alveolar defects and the patient's future restorative needs, immediate alveolar ridge preservation was performed using a bovine xenograft covered with a resorbable collagen membrane to reconstruct the extraction sites and maintain ridge volume for future implant placement. The surgical flaps were repositioned and sutured to obtain primary wound closure.

Postoperatively, the patient was prescribed amoxicillin 500 mg three times daily for seven days and ibuprofen 600 mg every eight hours for five days. Considering the high-energy mechanism of injury, the patient was immediately referred to the hospital for comprehensive medical evaluation, and neurological evaluation was also requested because of the facial and head trauma.

The patient returned one month after surgery for clinical follow-up. Soft tissue healing was satisfactory, with no signs of infection, wound dehiscence, or graft exposure. The sutures were removed, and the patient continued with periodic follow-up appointments. After four months of healing, clinical examination demonstrated satisfactory healing of the maxillary and mandibular edentulous ridges, with favorable soft tissue maturation and preservation of ridge architecture (Figure 2). Clinical and radiographic evaluation demonstrated ridge conditions considered adequate for implant placement.

**Figure 2:**
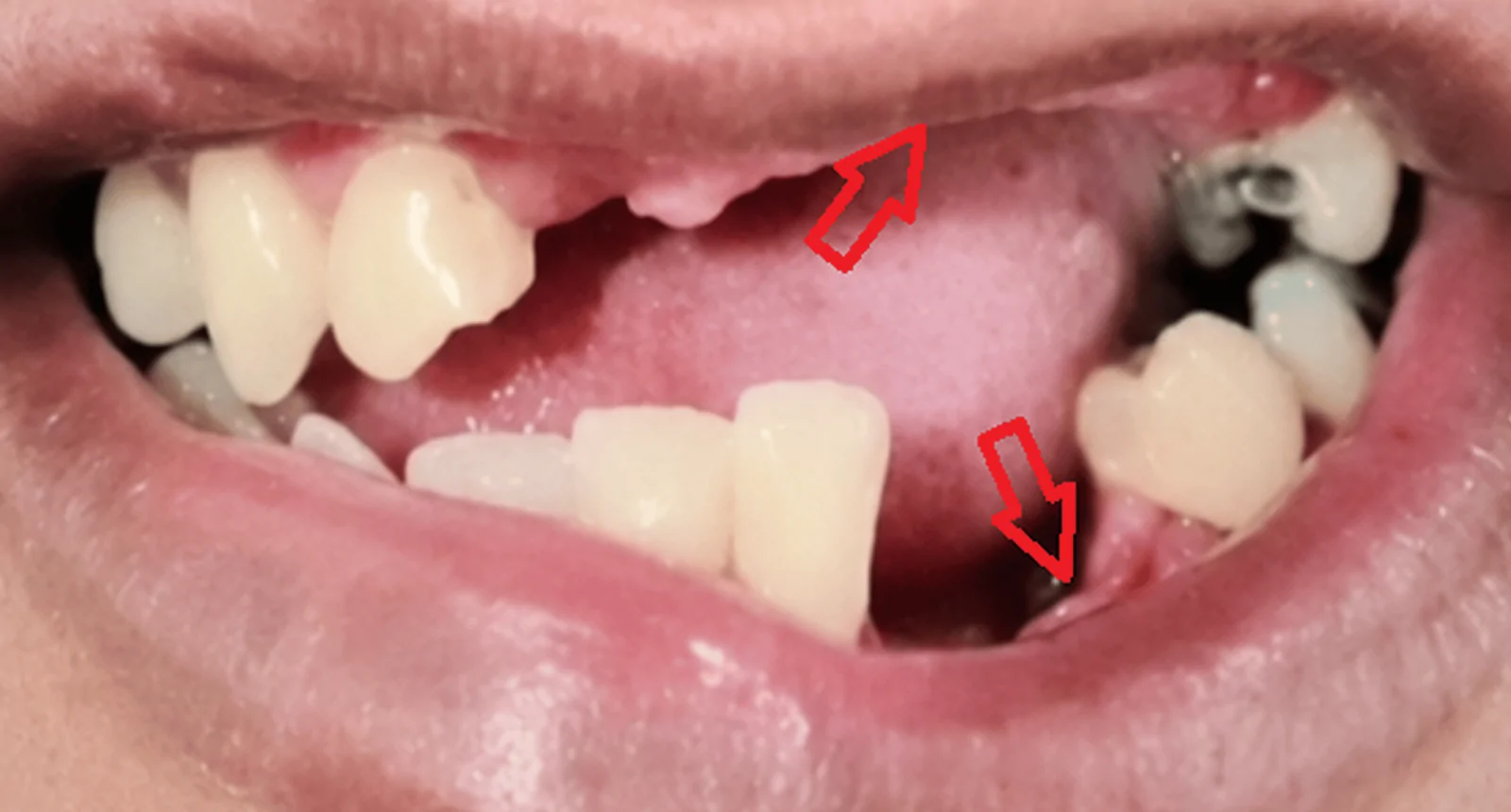
Clinical appearance of the alveolar ridges four months after ridge preservation. The clinical photograph demonstrates healed maxillary and mandibular edentulous ridges. The upper arrow indicates the maxillary alveolar ridge, and the lower arrow indicates the mandibular alveolar ridge.

Before implant surgery, the patient's systemic health was reassessed and remained classified as ASA I. Four dental implants were placed in the maxillary arch and two dental implants in the mandibular arch according to a prosthetically driven treatment plan. Following a six-month osseointegration period, clinical and panoramic radiographic evaluation demonstrated satisfactory implant integration and positioning of the four maxillary and two mandibular implants, supporting progression to the restorative phase (Figure 3).

**Figure 3:**
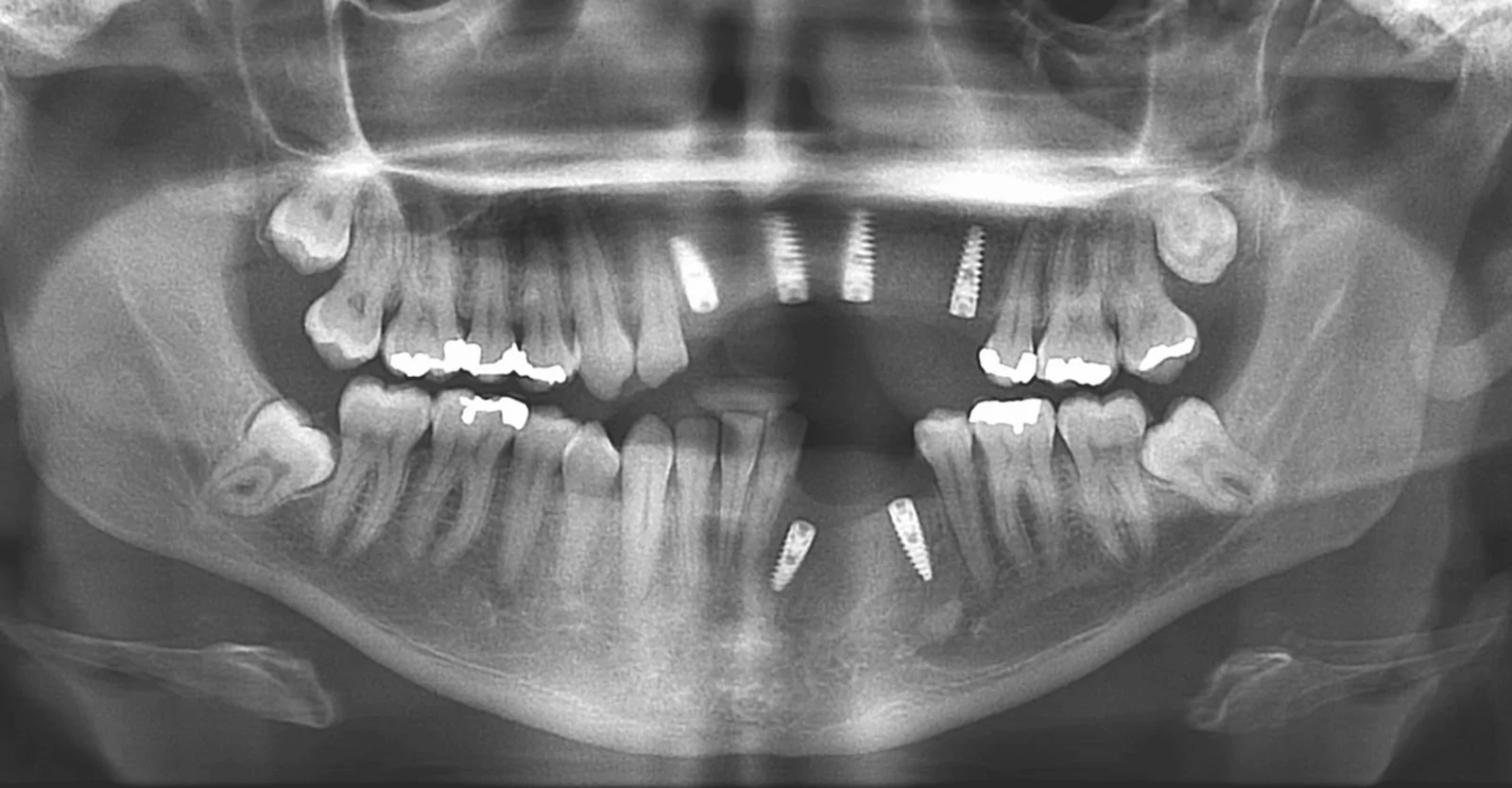
Panoramic radiographic evaluation following implant placement. Panoramic radiograph obtained after the six-month osseointegration period demonstrating the positioning of four maxillary and two mandibular dental implants.

A second-stage surgical procedure was subsequently performed, and healing abutments were placed for three weeks to allow peri-implant soft tissue maturation. After adequate soft tissue healing, scan bodies were positioned, and a digital impression was obtained for fabrication of the definitive implant-supported restorations. Clinical examination demonstrated favorable peri-implant soft tissue maturation around the maxillary and mandibular implant sites during the digital restorative workflow (Figure 4).

**Figure 4:**
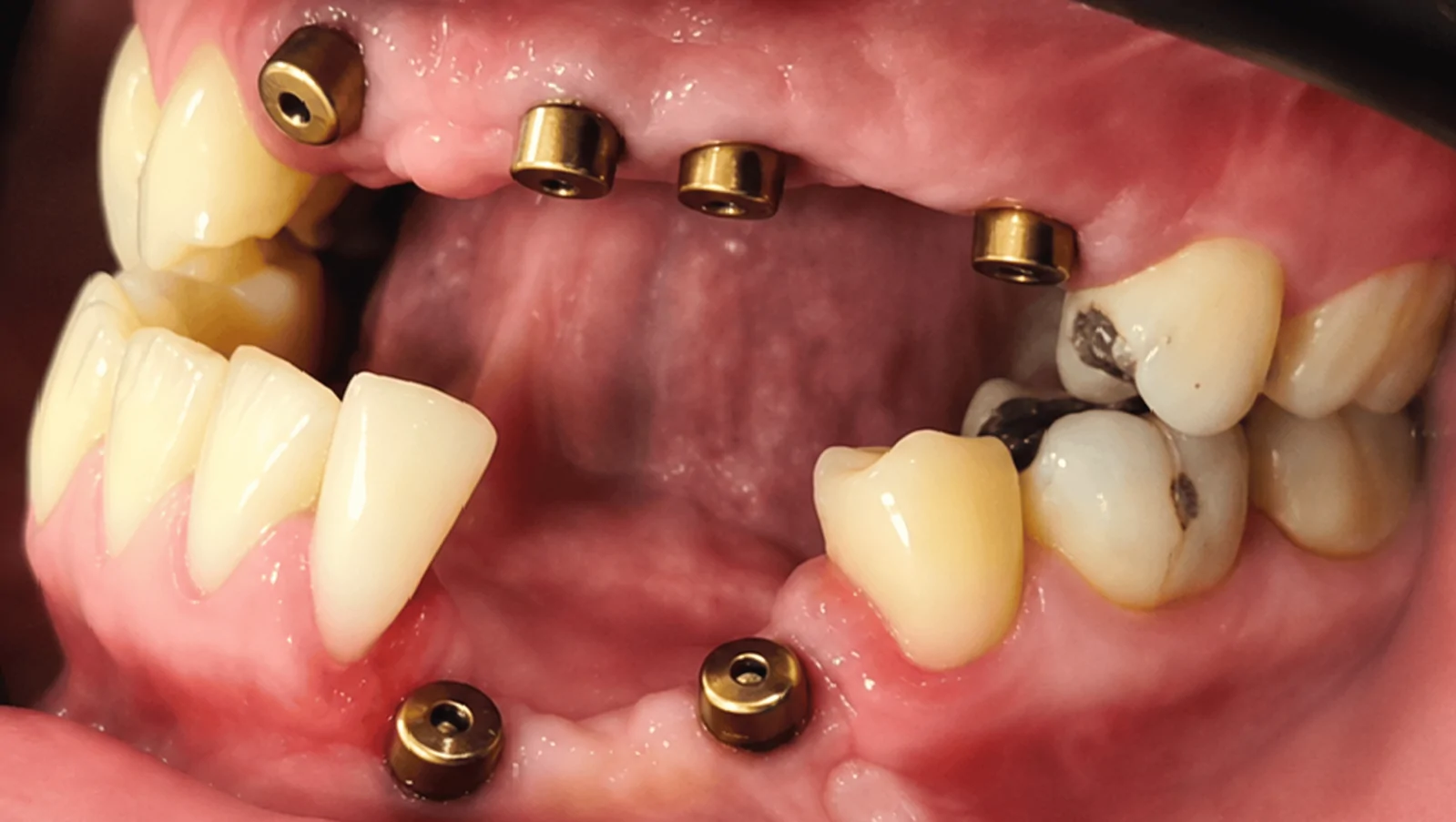
Digital restorative workflow following implant osseointegration. Clinical photograph demonstrating the maxillary and mandibular implant sites with scan bodies positioned during the digital restorative phase.

The digital impression was transmitted to the dental laboratory for fabrication of the definitive prostheses. Three weeks after the digital impression, the definitive implant-supported restorations were delivered. Clinical evaluation demonstrated satisfactory esthetic integration, favorable prosthetic contours, and restoration of the patient's smile and occlusal relationships (Figure 5).

**Figure 5:**
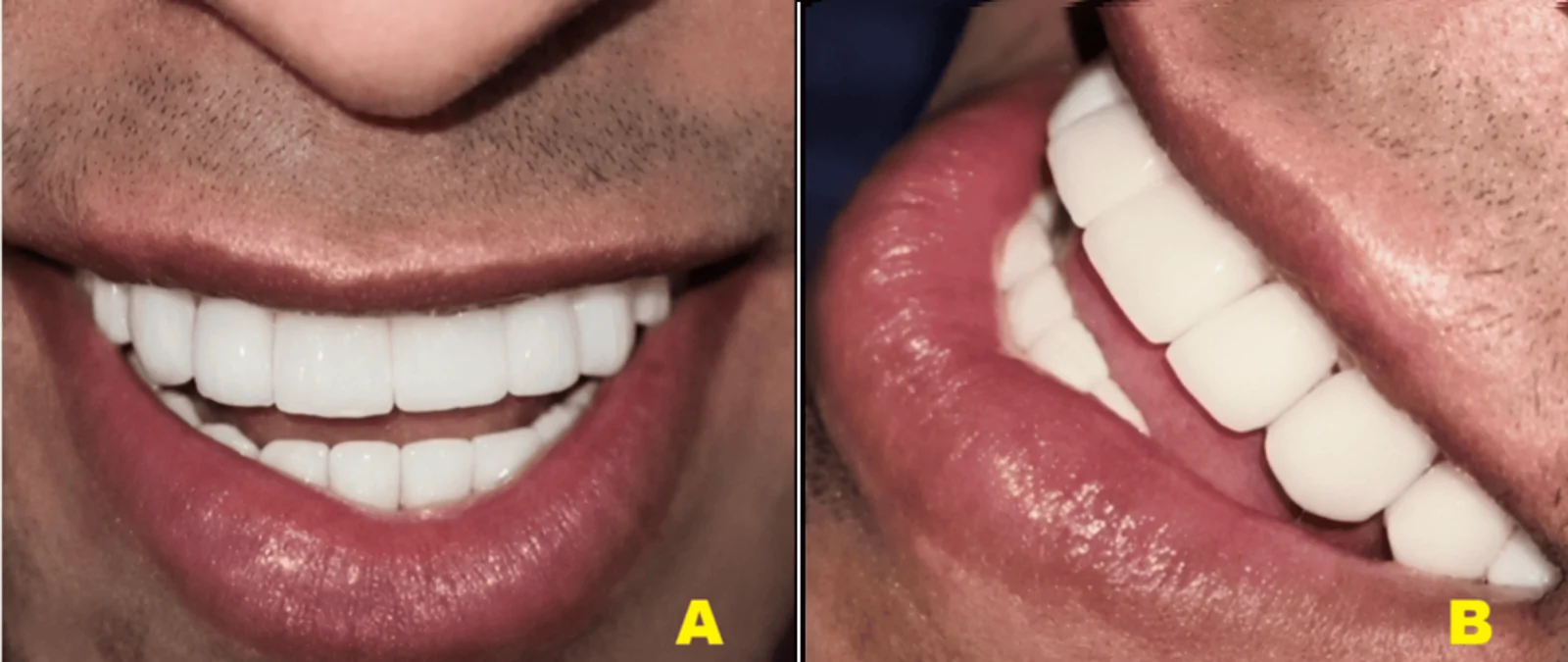
Final clinical outcome following implant-supported rehabilitation. Final clinical photographs following delivery of the definitive implant-supported restorations. (A) Frontal smile view demonstrating the definitive restorations. (B) Lateral smile view demonstrating the prosthetic contours, occlusal relationships, and surrounding soft tissues.

At the final clinical evaluation, the peri-implant soft tissues appeared stable, and the patient reported satisfaction with the functional and esthetic outcome. No biological or prosthetic complications were observed during the documented treatment period.

## Discussion

Severe dentoalveolar trauma caused by high-energy motorcycle accidents presents considerable clinical challenges because management extends beyond replacing missing teeth. Treatment requires preservation of the remaining alveolar structures, restoration of oral function and esthetics, and recognition of potentially serious injuries that may accompany facial trauma [1-4]. This case illustrates a staged approach to severe dentoalveolar trauma that emphasizes the management of traumatic dental injuries, preservation of the alveolar ridge, and subsequent implant-supported rehabilitation.

One of the most important aspects of this case was the immediate management of the traumatic extraction sites. Removal of non-restorable teeth, thorough irrigation of the surgical field, and conservative debridement of fractured and nonviable alveolar bone while preserving healthy bone whenever possible created favorable conditions for subsequent healing. Immediate alveolar ridge preservation using a bovine xenograft and a resorbable collagen membrane was performed to maintain ridge architecture following the traumatic extractions and facilitate subsequent implant placement [5-14]. In this case, ridge preservation was selected because of the traumatic alveolar defects and the anticipated need for future implant-supported rehabilitation. This approach should be considered according to the clinical characteristics of the traumatic sites and the patient's individual restorative needs rather than as a universal protocol for acute dentoalveolar trauma.

Another important consideration in this case was the recognition that injuries associated with a high-energy motorcycle accident may extend beyond the oral cavity. Although the patient presented primarily for dental treatment, hospital-based medical evaluation and neurological assessment were recommended because of the mechanism of injury and associated facial and head trauma. This case reinforces the importance of recognizing when additional medical evaluation is indicated in patients presenting with severe dentoalveolar trauma and of considering the patient's overall condition when planning subsequent dental rehabilitation [15-17].

Delayed implant placement following a four-month healing period allowed maturation of the preserved alveolar sites before implant placement. Following a six-month osseointegration period, clinical and panoramic radiographic evaluation supported progression to the restorative phase. Definitive implant-supported restorations were subsequently delivered to restore masticatory function, esthetics, and occlusal relationships. The clinical course observed in this patient supports consideration of a staged approach to implant rehabilitation following severe dentoalveolar trauma when ridge preservation and careful treatment planning are appropriate [18-20].

This report also highlights the importance of individualized treatment planning. The severity and pattern of traumatic injuries vary considerably among patients, and treatment decisions should consider the extent of alveolar injury, soft tissue condition, systemic health, future restorative requirements, and the possibility of associated facial or neurological injuries. The management described in this case represents an individualized approach and should not be interpreted as a standardized protocol for all patients with severe dentoalveolar trauma.

This report has limitations inherent to a single case. The findings cannot be generalized to all patients with dentoalveolar trauma. In addition, quantitative ridge measurements, standardized implant stability parameters, detailed peri-implant clinical measurements, and a precisely defined long-term post-restorative follow-up period were not available in the documented case information. These limitations restrict the objective assessment of the long-term treatment outcome. Nevertheless, the clinical course of this case illustrates how immediate ridge preservation followed by delayed implant placement and definitive implant-supported rehabilitation may provide useful clinical considerations for the staged management of selected patients with severe motorcycle-related dentoalveolar trauma.

## Conclusions

This case illustrates the staged management of severe dentoalveolar trauma following a high-energy motorcycle accident through careful assessment of the traumatic injuries, extraction of non-restorable teeth, preservation of viable alveolar structures, immediate ridge preservation, delayed implant placement, and definitive implant-supported rehabilitation. Following a four-month healing period and a six-month osseointegration period, the patient progressed to definitive rehabilitation with satisfactory functional and esthetic outcomes based on the documented clinical and radiographic findings.

Although this report describes a single patient, it provides clinical considerations that may assist clinicians when encountering similar severe dentoalveolar injuries. In patients presenting after high-energy trauma, clinicians should recognize that injuries may extend beyond the oral cavity and consider appropriate medical evaluation when associated systemic, craniofacial, or neurological injuries are suspected. Treatment decisions should be individualized according to the extent of the dentoalveolar injury, condition of the remaining hard and soft tissues, systemic considerations, and future restorative needs. The approach described in this case should therefore be interpreted as an individualized clinical strategy rather than a standardized treatment protocol.
